# Portable air cleaners should be at the forefront of the public health response to landscape fire smoke

**DOI:** 10.1186/s12940-016-0198-9

**Published:** 2016-11-25

**Authors:** Prabjit K. Barn, Catherine T. Elliott, Ryan W. Allen, Tom Kosatsky, Karen Rideout, Sarah B. Henderson

**Affiliations:** 1 Environmental Health Services, British Columbia Centre for Disease Control, 655 West 12th Avenue, Vancouver, BC V5Z 4R4 Canada; 2 National Collaborating Centre for Environmental Health, 200-601 West Broadway, Vancouver, BC V5Z 4C2 Canada; 3 Office of the Chief Medical Officer of Health, P.O. Box 2703, Whitehorse, Yukon Y1A 2C6 Canada; 4 Faculty of Health Sciences, Simon Fraser University, 8888 University Drive, Burnaby, BC V5A 1S6 Canada

**Keywords:** Landscape fire smoke, Wildfire smoke, Portable air cleaners, HEPA filters, Intervention

## Abstract

Landscape fires can produce large quantities of smoke that degrade air quality in both remote and urban communities. Smoke from these fires is a complex mixture of fine particulate matter and gases, exposure to which is associated with increased respiratory and cardiovascular morbidity and mortality. The public health response to short-lived smoke events typically advises people to remain indoors with windows and doors closed, but does not emphasize the use of portable air cleaners (PAC) to create private or public clean air shelters. High efficiency particulate air filters and electrostatic precipitators can lower indoor concentrations of fine particulate matter and improve respiratory and cardiovascular outcomes. We argue that PACs should be at the forefront of the public health response to landscape fire smoke events.

## Introduction

Fires in the landscape include both wildfires and controlled fires, which are used for fuel reduction, ecological restoration, land clearing, and agriculture. All landscape fires create smoke pollution and have the potential to affect human health, but most evidence on smoke exposures comes from research on wildfires. These fires, although episodic and generally short-lived, produce a substantial amount of air pollution. Their smoke is composed of fine particulate matter (PM_2.5_), carbon monoxide, nitrogen oxides, volatile organic compounds, as well as compounds such as polycyclic aromatic hydrocarbons and benzene [1]. Fine particulate matter is considered to be the most health damaging component of acute smoke exposures, which are typically characterized by monitored PM_2.5_ concentrations [1]. On very smoky days, 24-h average PM_2.5_ concentrations can be many times the World Health Organization guideline of 25 μg/m^3^. Smoke from landscape fires can affect communities in both rural and urban areas, sometimes hundreds of kilometers from the source. Additionally, exposures will become more prevalent as the global climate changes and wildfires become more frequent and intense [2].

Exposure to landscape fire smoke has important public health impacts. Mortality and respiratory morbidity have been the most frequently studied and most consistently reported outcomes of smoke exposure. Smoke-affected communities are at increased risk of all-cause mortality and respiratory-related emergency room and doctor visits, hospital admissions, and use of rescue medication [3, 4]. Less literature is available on cardiovascular outcomes, but recent evidence suggests that smoke exposure is also associated with increased cardiovascular mortality [5], hospital admissions for ischemic heart disease [6], and out-of-hospital cardiac arrests [3, 6]. Emerging evidence also links wildfire smoke to reduced birth weight, increased systemic inflammation, and bone marrow effects [3, 4].

Public health authorities are often required to respond to communities affected by wildfire smoke by providing general advice and/or recommending specific interventions. However, the public health response to smoke events has been constrained by limited evidence [1]. Five interventions have been commonly implemented: (i) evacuation to less smoky areas; (ii) providing information on N95 respirators; (iii) advising that people remain indoors and reduce physical activity; (iv) promoting the use of portable air cleaners (PACs) to create private or public clean air shelters in homes or large buildings with well-maintained ventilation systems; and (v) augmenting existing induct filtration for institutional settings [1]. Evacuations are stressful, and may not protect populations from smoke in the absence of direct threat from fire [7]. Respirator use is cumbersome and requires proper fit to be effective. The protection offered by staying indoors largely depends on building construction and the infiltration of outdoor air. Large indoor spaces may allow for dilution of smoke, and may have induct filtration as part of their ventilation systems. However, the utility of taking refuge in large indoor spaces for exposure reduction has not been evaluated during high pollution events [1]. On the other hand, a growing body of evidence suggests PACs are effective during such events [8].

We scanned the websites of eight North American public health authorities located in areas commonly impacted by wildfire smoke to review recommendations made during smoke events (Table 1). Although seven of the sites recommended PAC use, the level of information provided to the public varied widely. Among authorities recommending PAC use, advice ranged from specifying the type of air cleaner to use to simply stating “if you have room air cleaners, turn them on.” Few provided accompanying advice, such as the need to ensure proper sizing of the unit and limit airflow into the room or home. Public health recommendations on PAC use in both private and public clean air shelters should be stronger and provide specific guidance to maximize the effectiveness of this important intervention. Here we define private shelters as any building that would normally have restricted access (e.g. homes and residential care facilities), and public shelters as any building that would normally be accessible to the general public (e.g. community centers, shopping malls, and libraries).

**Table 1:** Recommendations made to the public regarding air cleaner use during wildfire smoke events

Public Health Authority	Recommendations^a^	
	Use a portable/room air cleaner	Type of air cleaner that should be used
*Canada*		
Alberta Health Services [23]	√	HEPA
Manitoba Health, Healthy Living and Seniors [24]	√	HEPA
Northwest Territories Health and Social Services [25]	√	HEPA
Ontario Ministry of Health and Long-Term Care [26]	√	HEPA
*United States*		
California EPA Air Resources Board [27]	X	X
Colorado Department of Public Health & Environment [28]	√	HEPA
North Carolina Public Health [29]	√	Mechanical air cleaners
Washington State Department of Health [30]	√	HEPA

^a^“√” indicates that information is provided and “X” indicates that no information is provided

In 2014, the British Columbia Centre for Disease Control published *Guidance for Public Health Decision Making During Wildfire Smoke Events*, which incorporated evidence reviews on the five most commonly implemented interventions listed above [1]. The aim of the document was to provide public health decision makers in British Columbia with a summary of the current evidence. Here we provide a synopsis of the evidence review on the use of PACs in private and public clean air shelters for a larger public health audience. Given the severity of recent wildfire seasons worldwide and the proven benefits of PAC use in high PM environments, we argue that PACs should be an integral part of the public health response to smoke events.

### Portable air cleaners

We define PACs as portable units equipped with high efficiency particulate air (HEPA) filters or electrostatic precipitators [8]. Portable units are designed to clean air in a single room, although some studies show reductions in whole house PM_2.5_ concentrations [9]. Both HEPA units and electrostatic precipitators target PM_2.5_: HEPA units use mechanical suction to pull air across a high efficiency filter, and electrostatic precipitators charge an incoming stream of particles and collect them on an oppositely charged metal plate. There has been limited evaluation of induct filters, but we include some discussion of their use.

### Portable air cleaners reduce indoor particulate matter

Two studies have assessed the impact of PAC use on residential indoor PM_2.5_ during wildfire events. Henderson et al. [9] compared indoor PM_2.5_ concentrations in two homes operating two or three electronic precipitators with two control homes that had no PACs. Sampling occurred over 24 to 48 h periods during wildfire events. Authors reported 63 to 88% lower PM_2.5_ concentrations in homes with PACs [9]. Barn et al. [10] investigated the impact of HEPA filters on infiltration efficiency, a measure of the contribution of outdoor PM_2.5_ to indoor concentrations. One PAC was operated in each home. Sampling occurred over consecutive 48-h periods when homes were impacted by smoke from wildfires or residential wood burning [10]. The study used a randomized crossover design, in which the HEPA filter was in place during one 24-h period and removed (control period) during the other 24-h period, with the order of filtration and control period selected randomly [10]. Among homes impacted by wildfire smoke (*n* = 13), a lower mean infiltration efficiency (± standard deviation) was found for filtration (19% ± 20%) compared with control (61% ± 27%) periods [10].

Most studies of PAC efficacy have assessed indoor PM_2.5_ from residential wood burning, traffic-related air pollution, or environmental tobacco smoke [8]. Many of these studies have used the same randomized design described above, which is considered the best way to evaluate interventions in health research. These studies show that PACs can reduce indoor PM_2.5_ concentrations by 32–88%, with most of this variability being attributed to differences in study design, including the number of units used, the study duration, and the airflow in the room/home.

Few studies have investigated PAC use in very high pollution settings. Chen et al. [11] reported a 57% reduction in indoor PM_2.5_ concentrations with HEPA filter PAC use in college dormitories located in Shanghai, China, where mean indoor concentrations were 96.2 (25.8) μg/m^3^ during the control period and outdoor concentrations were 102 (11.7) μg/m^3^. We are conducting a randomized controlled trial to assess HEPA filter use and fetal growth in Ulaanbaatar, Mongolia, where residential coal burning results in average wintertime outdoor PM_2.5_ concentrations of 250 μg/m^3^ [12]. Preliminary results show that PACs can reduce indoor PM_2.5_ concentrations by approximately 26%, on average (unpublished data).

### Potential health benefits

Two studies have investigated the potential health benefits of PAC use during wildfires. Mott et al. [13] examined respiratory symptoms and interventions among residents of the Hoopa Valley National Indian Reservation in California during a wildfire that was active from August 23 to November 3, 1999. Residents were surveyed about their respiratory symptoms before, during, and after the event, and about their use of four targeted interventions: portable HEPA filter air cleaners; public service announcements; face masks (N95 and non-filtered); and hotel vouchers to facilitate evacuation. The distribution of PACs and hotel vouchers was prioritized among those with pre-existing conditions. Ninety-eight participants (34%) reported using PACs for an average of 19.2 (95% CI: 17.8, 20.7) hours a day on 14.9 (95% CI: 11.8, 18.1) days during the smoke event. Increased duration of PAC use was associated with decreased odds of reporting worsening respiratory symptoms (OR = 0.54, confidence interval not reported). Null effects were found for mask use and evacuation [13]. Authors noted that approximately half of the PAC users operated the units during the 3 days when PM_10_ concentrations were highest, while only 17% of those using hotel vouchers had evacuated the area during this same period. The inability to take time away from work was reported as the main reason for residents choosing not to evacuate, but no other factors related to uptake or use of the interventions were reported. Findings from this study, although limited by recall bias and lack of exposure measurements, suggest that PACs may protect respiratory health during smoke events.

More recently, Fisk and Chan [14] modelled the potential health benefits and economic costs associated with filtration in homes during a 2003 wildfire smoke event that affected six counties in southern California. Health benefits were quantified as reductions in premature deaths and hospital admissions related to asthma, bronchitis, chronic obstructive pulmonary disease, and pneumonia. Cost estimates were based on purchase and operational costs associated with implementation of six intervention scenarios in (i) all homes, and (ii) homes of older adults (≥65 years) only, who were assumed to spend more time indoors at home compared with the general population. Three out of six intervention scenarios involved the use of PACs (as a stand-alone intervention or in conjunction with low and high efficiency induct filtration), with the remaining scenarios based on induct filtration only. Mean reductions in indoor PM_2.5_ concentrations of 45, 51 and 62% were estimated for PAC only use, PAC use with low efficiency induct filtration, and PAC use with high efficiency induct infiltration, respectively. In comparison, mean reductions for scenarios involving induct filtration ranged from 11 to 47%. The scenarios included continuous use of forced air systems with low efficiency filters (24%), continuous use of forced air systems with high efficiency filters (47%), and intermittent use of forced air systems with high efficiency filters (11%). The mean reduction in indoor PM_2.5_ concentrations by PACs alone was equivalent to that associated with continuous use of high efficiency induct filtration. During the wildfire period, 133 (95% CI: 26, 262) premature deaths and 417 (95% CI: 265, 655) respiratory hospital admissions were attributed to smoke among a total estimated population of 20.5 million. Model results estimated that use of PACs alone may have reduced smoke-related deaths and hospital admissions by 30 and 45%, respectively [14]. Targeted PAC use among older adults may have resulted in reductions of 50 and 78%, respectively [14]. Targeted implementation of PAC use among older persons was economically feasible, being associated with costs of $368 million (based 1.53 million homes and $239/PAC), and health-related benefits of $445 million (deaths and hospital admissions avoided) [14].

The benefits of PAC use for respiratory health are well-studied, with much of the literature suggesting their use in homes can improve asthma- and allergy-related symptoms [15]. Filtration efficiency of allergens may be greater because allergens tend to be larger than PM_2.5_ (in the 5 to 30 μm range). More recently, several studies have evaluated the effects of PAC use on cardiovascular health (Table 2). These studies have mostly used randomized designs to evaluate objective health outcomes. To date, PAC use has been linked to improvements in cardiovascular indicators such as endothelial function [16, 17], systemic inflammation [11, 17], and blood pressure [11, 18]. These studies report benefits over 2–7 days of PAC use, the typical duration of smoke episodes. The mean and median baseline PM_2.5_ concentrations were low (≤ 8 μg/m^3^) in two studies that reported null associations [19, 20]. Another study reported higher blood concentrations of IL-6, a marker of inflammation, with PAC use [21]. The filtration (PAC use) and control (no PAC use) periods were randomly assigned for each participant. The authors noted that baseline differences in IL-6 concentrations and use of anti-inflammatory medication between groups who received the intervention in the first half versus the second half of the study could in part explain these findings [21].

**Table 2:** Summary of studies investigating portable air cleaner (PAC) use and indicators of cardiovascular health

Authors (year)	Type of PAC and study design	Exposure, outcome^a^, location, and study population	Main findings
Brauner et al. [16]	HEPA filter Two PACs were operated in each home for 4 days. Units were randomly operated with the filter (filtration period) and without the filter (control period) each for half the study period.	Traffic related PM_2.5_ Endothelial function (RHI) Copenhagen, Denmark Older adults aged 60–75 years; *n* = 45	Exposure: Indoor PM_2.5_ concentrations were reduced by 62%. Geometric mean indoor PM_2.5_ concentrations were 4.7 μg/m^3^ (95% confidence interval (CI): 3.9, 5.7 μg/m^3^) during the filtration period and 12.6 μg/m^3^ (95% CI: 11.2, 14.1 μg/m^3^) during the control period. Health: RHI increased by 8.1% (95% CI: 0.4, 16.3%).
Allen et al. [17]	HEPA filter Two PACs were operated in each home for 14 days. Units were randomly operated with the filter (filtration period) and without the filter (control period) each for half the study period.	Wood smoke related PM_2.5_ Endothelial function (RHI); inflammation (CRP) Northern British Columbia, Canada Adults aged 20–63 years; *n* = 45	Exposure: Indoor PM_2.5_ concentrations were reduced by 59%. Mean (standard deviation) indoor PM_2.5_ concentrations were 4.6 (2.6) μg/m^3^ during the filtration period and 11.2 (6.1) μg/m^3^ during the control period. Health: RHI increased by 9.4% (95% CI: 0.9, 18%) and CRP decreased by 32.6% (95% CI: 4.4, 60.9%).
Weichenthal et al. [18]	Electrostatic precipitator One PAC was operated in each home for 14 days. Units were randomly operated with the precipitating plates (filtration period) and without the plates (control period) each for half the study period.	Indoor PM_2.5_ (no specific source) Blood pressure Manitoba, Canada Children and adults aged 11–64 years; *n* = 37	Exposure: Indoor PM_2.5_ concentrations were reduced by 52%. Mean (standard deviation) indoor PM_2.5_ concentrations were 30.0 (30) μg/m^3^ during the filtration period and 61.0 (64) μg/m^3^ during the control period. Health: Systolic blood pressure decreased by 7.9 mm Hg (95% CI: −17, 0.82 mm Hg) and diastolic blood pressure decreased by 4.5 mm Hg (95% CI: −11, 2.4 mm Hg).
Karottki et al. [19]	Electrostatic precipitator Two PACs were operated in each home for 14 days. Units were randomly operated with the precipitating plates (filtration period) and without the plates (control period) each for half the study period.	Traffic related PM_2.5_ Endothelial function (RHI); inflammation (CRP); blood pressure Greater Copenhagen, Denmark Older adults aged 51–81 years; *n* = 48	Exposure: Indoor PM_2.5_ concentrations were reduced by 46% overall, but large variations in efficacy were seen within and across homes. Median (5th–95th percentile) indoor PM_2.5_ concentrations were 4.3 μg/m^3^ (0.2–12.2) during the filtration period and 8.0 μg/m^3^ (3.4–20.7) during the control period. Health: No significant effects.
Chen et al. 2015 [11]	Electrostatic precipitator One PAC was operated in each room (in college dormitory) for two 48 h periods, which were separated by a 2 week washout period. Units were randomly operated with the filter (filtration period) and without the filter (control period) each for one of the 48 h periods.	Indoor PM_2.5_ (no specific source) Shanghai, China Inflammation (CRP, fibrinogen, P-selectin, MCP-1, IL-1β, TNF-α, IL-6, MPO); blood coagulation (sCD4OL, PAI-1, t-PA, D-dimer); vasoconstriction (ET-1, ACE); blood pressure College students with a mean (standard deviation) age of 23 (2) years; *n* = 35	Exposure: Indoor PM_2.5_ concentrations were reduced by 57%. Mean (SD) indoor PM_2.5_ concentrations were 41.3 (17.6) μg/m^3^ during the filtration period and 96.2 (25.8) μg/m^3^ during the control period. Health: Geometric mean concentrations of several markers of inflammation and coagulation were reduced: MCP-1 by 17.5% (95% CI: 5.5, 30.8%), IL-1β by 68.1% (95% CI: 44.3, 81.7%), MPO by 32.8% (95% CI: 5.3, 67.5%), and sCD4OL by 64.9% (95% CI: 30.3, 82.3%). Geometric mean systolic and diastolic blood pressure decreased by 2.7% (95% CI: 0.4, 5.1%) and 4.8% (95% CI: 1.2, 8.5%), respectively.
Kajbafzadeh et al. [20]	HEPA filter Two PACs were operated in each home for 14 days. Units were randomly operated with the filter (filtration period) and without the filter (control period) each for half the study period.	Wood smoke or traffic-related PM_2.5_ Endothelial function (RHI); inflammation (CRP, IL-6) Greater Vancouver area, Canada Adults aged 19–72 years; *n* = 83	Exposure: Indoor PM_2.5_ concentrations were reduced by 40%. Mean (standard deviation) indoor PM_2.5_ concentrations were 4.3 (3.7) μg/m^3^ during the filtration period and 7.1 (6.1) μg/m^3^ during the control period. Health: No significant effects.
Padro-Martinez et al. [21]	HEPA filter (window unit) Two PACs were operated in each home for 42 days. Units were randomly operated with the filter (filtration period) and without the filter (control period) each for half the study period.	Traffic-related ultrafine particles (< 100 nm) Inflammation (IL-6, CRP, fibrinogen, TNF-RII); blood pressure Somerville, Massachusetts, United States Older adults with mean (standard) age of 53.6 (9.2) years; *n* = 20	Exposure: Particle counts were reduced by 45% (ranged from 0 to 68%). Health: IL-6 concentrations were on average 49.6% (95% CI: 5.90, 93.3%) lower during the control period compared with the filtration period.

^a^ *RHI* reactive hyperemia index, *CRP* c-reactive protein, *MCP* monocyte chemoattractant protein, *IL* interleukin, *TNF* tumor necrosis factor, *MPO* myeloperoxidase, *sCD40L* soluble CD40 ligand, *PAI* plasminogen activator inhibitor, *t-PA* tissue plasminogen activator, *ET-1* endothelin-1, *ACE* angiotensin-converting enzyme

### Implementing PAC use

Unfortunately, very little has been published on the considerations and challenges of implementing widespread PAC use during smoke events, and more evidence would benefit the public health community. Regardless, several considerations are likely required for successful implementation of the intervention, including: the intensity and duration of the smoke event; timing and preparation for implementation; costs, availability, and accessibility of the units; public health messaging; and needs of the community. Smoke from landscape fires is unpredictable, and timely management of exposures may require a large number of units over a short time period. Additionally, the costs of purchasing PACs may be prohibitive unless subsidized or provided free-of-charge to community members. Public health authorities may want to consider targeted implementation of PAC use among susceptible sub-populations in their primary response, but may need to expand the response to the general public for longer and/or more intense smoke events. Susceptible groups include people with pre-existing conditions, pregnant women, infants, children, older adults, and people of lower socioeconomic status [3, 14]. Guidance on effective PAC use should include information on types of units, appropriate sizing, limiting ventilation to reduce entry of outdoor air, and potential risks from heat and indoor-generated pollutants. The Clean Air Delivery Rate (CADR) is a voluntary industry rating system developed for portable HEPA filter devices that can provide guidance on appropriate sizing. The CADR ratings are listed on some units, and describe their efficiencies at different room sizes based on three pollutants: tobacco smoke, dust, and pollen [22]. The CADR rating for tobacco smoke is most relevant to wildfire smoke [22].

The use of both private and public clean air shelters should be considered in the public health response to landscape fire smoke. In addition to staying indoors in homes, community members may also be advised to spend time in designated public clean air shelters, such as shopping malls and libraries, which ideally limit the entry of outdoor air and allow for dilution of smoke. Taking advantage of existing induct filtration or air conditioning in large buildings is a practical approach, particularly when these systems use high efficiency filtration. However, some buildings may only be equipped to operate low efficiency filters that provide limited benefits with respect to exposure reduction. In such situations, PACs can offer a flexible solution to temporarily augment existing low efficiency induct filtration systems, given that portable air cleaners are adequately sized to clean these spaces. The ability of individuals to access public clean air shelters should also be considered. Although these shelters can provide benefits to larger populations, travel to these spaces may be difficult for some, particularly during smoky conditions, and time spent there is typically intermittent. On the other hand, filtration in residential buildings can offer benefits that are more continuous because people may spend more time in private clean air shelters than in public clean air shelters, especially those at highest risk. The continued evaluation of PAC use during wildfires in private and public spaces could help to establish best practice guidelines.

## Conclusions

Landscape fires can expose communities to high concentrations of PM_2.5_ and other pollutants. Many well-conducted studies show that PACs can lower indoor PM_2.5_ exposures and benefit respiratory and cardiovascular health. Public health officials should promote PAC use as a fundamental part of their response to smoke from landscape fires.

## Abbreviations

CADR: Clean air delivery rate
HEPA: High efficiency particulate air
PAC: Portable air cleaner
